# Analysis of risk factors for the recurrence of osteomyelitis of the limb after treatment with antibiotic-loaded calcium sulfate and autologous bone graft

**DOI:** 10.3389/fbioe.2024.1368818

**Published:** 2024-05-14

**Authors:** Yu Su, Dongchen Li, Bing Du, Zhao Li, Yao Lu, Yibo Xu, Qian Wang, Zhong Li, Cheng Ren, Teng Ma

**Affiliations:** Honghui Hospital, Xi’an Jiaotong University, Xi’an, Shaanxi, China

**Keywords:** calcium sulfate, osteomyelitis, recurrence of infection, autogenous iliac bone, analysis of risk factors

## Abstract

**Objective:**

We aimed to evaluate the efficacy of antibiotic-loaded calcium sulfate combined with autologous iliac bone transplantation in the treatment of limb-localized osteomyelitis (Cierny–Mader type III) and analyze the causes and risk factors associated with infection recurrence.

**Methods:**

Clinical data of 163 patients with localized osteomyelitis of the extremities treated with antibiotic-loaded calcium sulfate combined with autologous iliac bone transplantation in Xi’an Honghui Hospital from January 2017 to December 2022 were retrospectively analyzed. All patients were diagnosed with localized osteomyelitis through clinical examination and treated with antibiotic-loaded calcium sulfate combined with autologous iliac bone. Based on the infection recurrence status, the patients were divided into the recurrence group and the non-recurrence group. The clinical data of the two groups were compared using univariate analysis. Subsequently, the distinct datasets were included in the binary logistic regression analysis to determine the risk and protective factors.

**Results:**

This study included 163 eligible patients, with an average age of 51.0 years (standard deviation: 14.9). After 12 months of follow-up, 25 patients (15.3%) experienced infection recurrence and were included in the recurrence group; the remaining 138 patients were included in the non-recurrence group. Among the 25 patients with recurrent infection, 20 required reoperation, four received antibiotic treatment alone, and one refused further treatment. Univariate analysis showed that education level, smoking, hypoproteinemia, open injury-related infection, and combined flap surgery were associated with infection recurrence (*p* < 0.05). Logistic regression analysis showed that open injury-related infection (odds ratio [OR] = 35.698; 95% confidence interval [CI]: 5.997–212.495; *p* < 0.001) and combined flap surgery (OR = 41.408; 95% CI: 5.806–295.343; *p* < 0.001) were independent risk factors for infection recurrence. Meanwhile, high education level (OR = 0.009; 95% CI: 0.001–0.061; *p* < 0.001) was a protective factor for infection recurrence.

**Conclusion:**

Antibiotic-loaded calcium sulfate combined with autologous iliac bone transplantation is an effective method for treating limb-localized osteomyelitis. Patients without previous combined flap surgery and non-open injury-related infections have a relatively low probability of recurrence of infection after treatment with this surgical method. Additionally, patients with a history of smoking and hypoproteinemia should pay attention to preventing the recurrence of infection after operation. Providing additional guidance and support, particularly in patients with lower education levels and compliance, could contribute to the reduction of infection recurrence.

## 1 Introduction

Osteomyelitis is an inflammatory bone disease caused by infection, which can easily lead to necrosis and destruction of bone tissue. It is one of the most challenging musculoskeletal complications of traumatic orthopedic surgeries (Rao et al., 2011; Metsemakers et al., 2018). In recent years, the prevalence of osteomyelitis has steadily increased due to several factors such as advancements in diagnostic capabilities, increased incidence of trauma, inappropriate use of implants, or occurrence of bloodstream infections (Walter et al., 2021). Concurrently, bacterial virulence and autoimmunity contribute to the difficulty of controlling infections or result in a high recurrence rate, presenting an ongoing concern for orthopedic surgeons (Wang et al., 2020). Once osteomyelitis manifests, more severe complications develop, including nonunion, pathological fractures, and amputation, significantly diminishing patients’ quality of life (Zhou et al., 2020). Despite standardized treatment protocols, the recurrence rate of chronic osteomyelitis remains high (20%–30%) (Zhou et al., 2020).

In 1985, Cierny and Mader introduced a new classification system for osteomyelitis to describe the degree of inflammation and improve the therapeutic effect. According to anatomical bone involvement, osteomyelitis is divided into four types: medullary type (Type I), superficial type (Type II), localized type (Type III), and diffuse type (Type IV). Furthermore, patients are divided into three groups based on their physiological condition as follows: healthy patients (Group A), damaged patients (Group B), and frail patients who cannot undergo surgery (Group C). This classification has since been widely acknowledged as the standard classification of long bone osteomyelitis (Cierny et al., 2003). Compared with severe diffuse osteomyelitis (Cierny–Mader [C-M] type IV), localized osteomyelitis (type III) has a limited infection range, milder clinical symptoms, and lower treatment complexity. Consequently, prompt and accurate surgical intervention can yield superior clinical outcomes. Traditional treatment typically involves a staged approach, beginning with antibiotic chain bead implantation followed by cancellous bone transplantation after achieving infection control (Emami et al., 1995). In 1986, Masquelet introduced a two-stage technique for repairing large bone defects. In 2000, he proposed the concept of an induced membrane based on his case results (Masquelet et al., 2000). Subsequent studies have demonstrated the efficacy of this technique in the treatment of osteomyelitis, establishing it as a notable representative of staged treatment due to its simplicity and low infection rate (Tong et al., 2017; Wang et al., 2020; Shi et al., 2023; Zhang et al., 2023). However, its drawbacks include the need for multi-stage and complex surgical management, prolonged systemic antibiotic therapy leading to potential damage to the metabolic organs, and the risk of donor complications, all of which increase the patients’ economic burden and psychological stress. To address these challenges, topical antibiotic-loaded bone substitute material implantation has emerged as an effective method (Batista Campos et al., 2023). Calcium sulfate has been widely used in orthopedic trauma in recent years owing to its excellent biocompatibility, degradability, and bone conductivity (Zhou et al., 2020; Batista Campos et al., 2023). When implanted into the lesion as an antibiotic-impregnated material, calcium sulfate offers numerous benefits, including versatility in drug loading, stable degradation rate, high local antibiotic concentration, and minimal side effects (Batista Campos et al., 2023; Domenicucci et al., 2023; Du et al., 2023). With the advancements in surgical debridement and microsurgical techniques, one-stage treatment of localized osteomyelitis has been achieved (Ruan et al., 2021). Previous studies have shown that antibiotic-loaded calcium sulfate can yield favorable clinical outcomes in the treatment of localized osteomyelitis of the tibia (Zhou et al., 2020).

In the radical treatment of osteomyelitis, recurrence is the primary concern among patients. However, despite the increasing incidence of osteomyelitis, only a few studies have examined the risk factors of antibiotic-loaded calcium sulfate in the treatment of localized osteomyelitis recurrence or reinfection. In this study, the data of 163 patients who met the inclusion and exclusion criteria were retrospectively analyzed. We aimed to determine the efficacy of antibiotic-loaded calcium sulfate combined with autologous iliac bone transplantation in the treatment of localized osteomyelitis of the extremities, and to identify the causes and risk factors of infection recurrence. The study results will provide clinical guidance for reducing the occurrence of such complications.

## 2 Materials and methods

### 2.1 Inclusion and exclusion criteria

**FIGURE 1:**
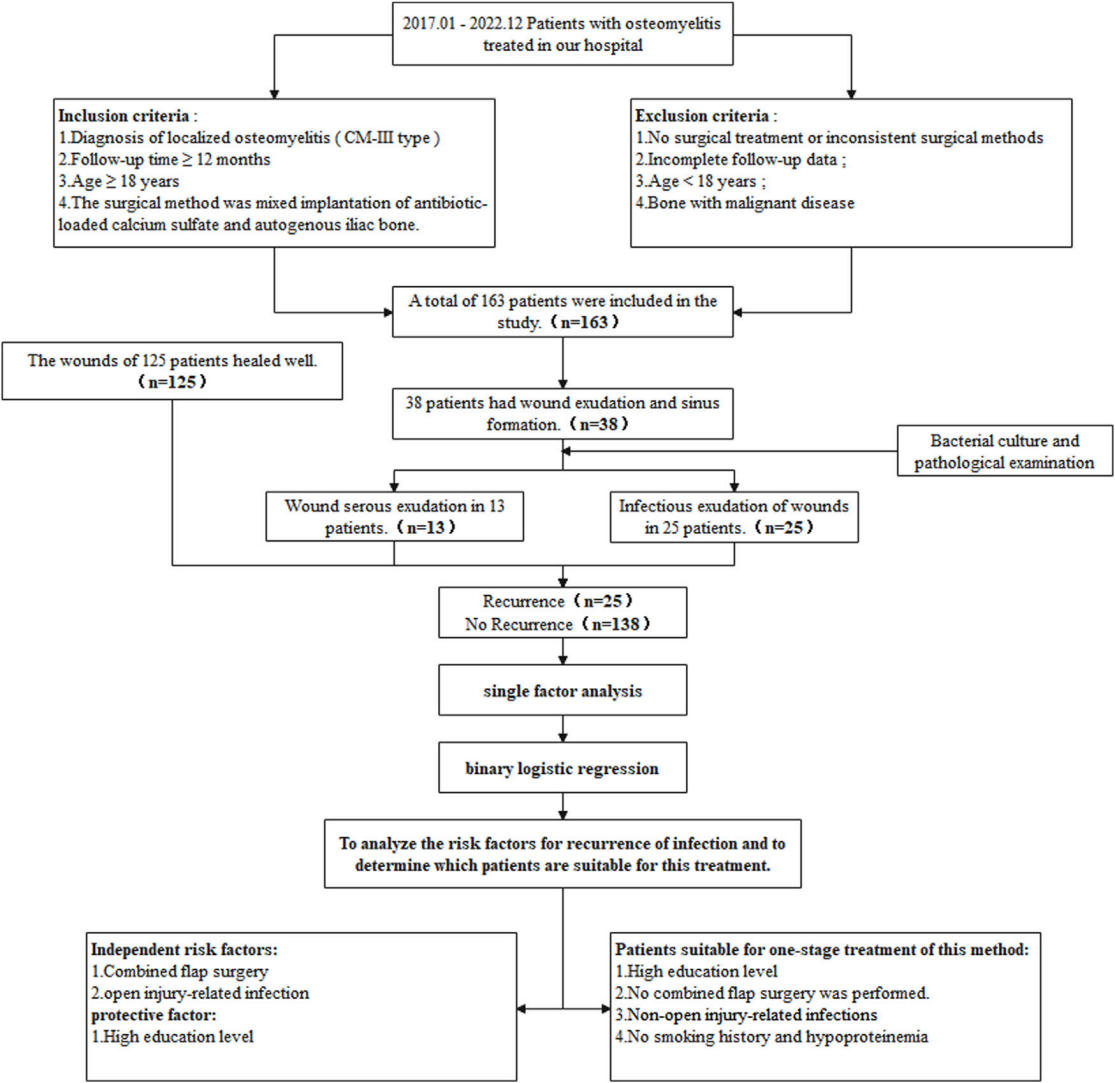
Flow chart showing the research ideas and results of this study.

This study included 163 patients with limb-localized osteomyelitis who were treated with antibiotic-loaded calcium sulfate combined with autologous iliac bone transplantation in our hospital from January 2017 to December 2022. Two experienced orthopedic surgeons performed the C-M classification on the patients, and a third surgeon was tasked to evaluate the decisions. This study was approved by the Ethics Committee of Xi’an Red Cross Hospital, and all patients signed an informed consent form. The flow chart of this study is shown in Figure 1.

Inclusion criteria were as follows: Patients who (Rao et al., 2011) were diagnosed with localized osteomyelitis of the extremities (C-M type III) based on the clinical manifestations, laboratory examination results, and imaging findings (Metsemakers et al., 2018); were followed up for ≥12 months (Walter et al., 2021); were aged ≥18 years; and (Wang et al., 2020) underwent antibiotic-loaded calcium sulfate treatment combined with autologous iliac bone transplantation after thorough debridement.

Exclusion criteria were as follows: Patients (Rao et al., 2011) who did not undergo any surgical treatment or other surgical methods (Metsemakers et al., 2018); with incomplete follow-up data (Walter et al., 2021); aged <18 years; and (Wang et al., 2020) who developed malignant diseases.

### 2.2 Operative procedure

Thorough debridement: All surgical procedures were performed under nerve block combined with general anesthesia, using an upper airbag tourniquet. Based on the site of infection and preoperative imaging results, an incision was made along the wound or the original surgical incision. The sinus tract and the surrounding bone scar were excised, and the internal fixation materials were exposed and removed. The intramedullary pus, inflammatory granulation tissue, and scar tissue were cleared; the dead bone was extracted using a bone rongeur; and the hardened tissue was removed with a high-speed grinding drill until punctate bleeding was observed at the fracture end and within the soft tissue (Paprika sign), indicating thorough debridement and adequate local blood supply. After debridement, the wounds were cleansed thrice with 3% hydrogen peroxide, normal saline, and iodophor stock solution. During surgery, at least four specimens (including pus, inflammatory granulation tissue, and necrotic bone) were collected from different sites for bacterial culture and histopathological examination. The size of the residual defect after debridement was measured. Surgical instruments were replaced, and the sheets were repositioned after a local disinfection.

Defect filling: Based on the size of the defect, an appropriate amount of autologous iliac bone was harvested from the iliac plate and processed into 3 mm × 3 mm × 3 mm particles. According to the results of the preoperative bacterial culture, either vancomycin or gentamicin powder was thoroughly mixed with calcium sulfate powder at a predetermined proportion in a sterile mixing bowl (or mixed solely with calcium sulfate powder for those with a negative bacterial culture result), and an appropriate amount of injectable water was added. After thorough mixing, the mixture was molded into granules using a silica gel mold. The antibiotic-loaded calcium sulfate and the iliac bone particles were combined at a 1:3 volume ratio. The mixture was then implanted into the cavity after it had completely solidified. Before wound closure, a negative pressure drainage tube was inserted to ensure smooth drainage. Patients with unstable bone structures underwent stabilization using an Ilizarov external fixator. Wounds with minimal tension were sutured directly, whereas those with significant tension or large skin defect areas underwent flap transfer to cover the wound.

### 2.3 Infection recurrence and treatment

The following criteria were employed to assess the possibility of infection recurrence: redness, swelling, heat, pain, and other symptoms in the primary site; increased body temperature; elevated levels of inflammatory markers on blood routine examination; and recurrence of sinus tract or infectious exudation in and around the wound. Distinguishing infectious exudation from serous exudation is crucial. Infectious exudation serves as a pivotal indicator for diagnosing infection recurrence. However, individuals who are unfamiliar with the characteristics of serous exudation may misdiagnose this condition as infectious exudation, potentially leading to unnecessary treatment interventions. Serous exudation occurs as a component of the slurry discharged from the wound during the absorption of calcium sulfate. Typically, this condition resolves naturally within 8 weeks without necessitating secondary surgical treatment. By contrast, infectious exudation commonly manifests as yellow-white pus, which can be accompanied by bloody exudation and a foul odor. Meanwhile, serous exudation primarily presents as light yellow exudates. Furthermore, infectious exudation is frequently associated with alterations in appearance and abnormalities in the levels of inflammatory indicators detected through laboratory testing; bacterial culture and pathological examination are also important bases for diagnosis. In patients with suspected false-negative or false-positive results on bacterial culture, further metagenomic testing can be performed to confirm the diagnosis.

Treatment after infection recurrence: When signs of infection recurrence were observed, sensitive antibiotics were administered intravenously based on the initial bacterial culture results until the symptoms improved. If no remission was observed or if symptoms worsened within 2 weeks, antibiotic-loaded calcium sulfate combined with autologous iliac bone transplantation was performed after debridement.

### 2.4 Observed indexes

The following demographic data of patients were collected from the electronic medical records: age, sex, education level, smoking history, comorbidities (diabetes, heart disease, hypertension, and hypoproteinemia), previous surgery (≥2 times), open injury-related infection, infection site, bacterial species, and combined flap surgery. Followup was conducted through outpatient review, WeChat messaging, or telephone consultations.

### 2.5 Statistical analysis

Statistical Package for Social Sciences (SPSS for Windows, version 22.0, Chicago, SPSS Inc.) was used to analyze data. Univariate analysis was conducted on variables that could contribute to the recurrence of infection, including age, sex, education level, smoking history, comorbidities (diabetes, heart disease, hypertension, and hypoproteinemia), previous surgery (≥2 times), open injury-related infection, infection site, bacterial species, and combined flap surgery. The measurement data were expressed as the mean ± standard deviation and analyzed using the normality test and two independent samples *t*-test. The count data were expressed as cases and percentages (%) and analyzed using the chi-square or Fisher’s exact test. Variables with significant differences in the univariate analysis were included in the multivariate logistic regression models. Binary logistic regression analysis was used to evaluate the independent association between these factors and infection recurrence. Statistical significance was set at a 5% level (*p* < 0.05) for all analyses.

## 3 Results

According to the predefined inclusion and exclusion criteria, a total of 163 eligible patients were identified and enrolled in this study. Among them, 136 were men, while 27 were women, with an average age of 51.0 years (range: 18–85 years). In terms of educational attainment, 66, 67, and 30 participants had completed primary school or a lower education level, secondary education, and university education or a higher education level, respectively. Additionally, 68 people patients had a history of smoking. In terms of comorbidities, 49 patients had diabetes, 17 had heart disease, 30 had hypertension, and 53 had hypoproteinemia. Furthermore, 32 patients had previously undergone surgery two or more times, while 91 patients developed open injury-related infection. The affected sites included tibia (84 patients), femur (39 patients), calcaneus (15 patients), fibula (7 patients), patella (10 patients), humerus (6 patients), and ulna and radius (2 patients). Pathogenic bacteria were not detected in six patients. Meanwhile, *Staphylococcus aureus* was detected in 82 patients, methicillin-resistant *Staphylococcus aureus* in 22 patients, *Pseudomonas aeruginosa* in 34 patients, *Escherichia coli* in eight patients, and *Staphylococcus epidermidis* in 11 patients. Additionally, 45 patients underwent combined flap surgery.

### 3.1 Characteristics and treatment of infection recurrence

**FIGURE 2:**
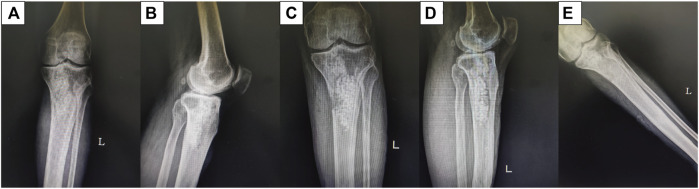
Male patient aged 43 years. Radiographs obtained before and after treatment with antibiotic-loaded calcium sulfate combined with autogenous iliac bone transplantation to manage proximal tibial osteomyelitis. **(A, B)** Preoperative anteroposterior and lateral position. **(C, D)** Anteroposterior and lateral position 1 d after the surgery. **(E)** Twelve months after the surgery.

A total of 163 patients were treated with antibiotic-loaded calcium sulfate combined with autologous iliac bone transplantation. Based on outpatient reviews and 12-month follow-up data, 125 patients exhibited satisfactory wound healing after surgery without any notable abnormalities, as illustrated in Figure 2. Wound fluid exudation and sinus formation occurred in 38 patients. The exudate was subjected to bacterial culture and pathological examination, and blood routine and biochemical examinations were performed. Thirteen patients showed negative bacterial culture results, with the exudate appearing light yellow and bright. Additionally, no signs of redness, swelling, heat, and pain were noted around the wound. Combined with a routine blood test, no abnormalities were found in the infection indicators. Hence, the condition was considered a serous exudation caused by calcium sulfate absorption and therefore excluded from the study. The remaining 25 patients exhibited elevated local skin temperature and yellowish-white purulent secretions. Bacterial culture showed positive and negative results in 20 and five patients, respectively. Further metagenomic detection yielded positive and negative results in three and 2, respectively. Considering that most patients had used antibiotics before bacterial culture, potentially affecting the examination results, along with the presence of clinical manifestations, these two patients were also diagnosed with infectious exudation and therefore included in the study. After excluding 13 patients with serous exudation, only 25 patients with postoperative infection recurrence were analyzed.

Among 25 (15.3%) patients with recurrent infection, four were successfully treated with intravenous antibiotics for only 2 weeks. Twenty patients underwent redebridement and reimplantation of antibiotic-loaded calcium sulfate combined with autologous iliac bone to manage the infection, with no recurrence observed within 12 months. One patient refused further treatment, expressing satisfaction with the current treatment outcomes due to low physical demand.

### 3.2 Analysis of risk factors for recurrence of infection

First, univariate analysis was performed to assess the association between potential factors and infection recurrence. The results were as follows (Table 1): age (*p* = 0.630), sex (*p* = 1.000), diabetes (*p* = 0.239), heart disease (*p* = 1.000), hypertension (*p* = 1.000), previous surgery (≥2 times) (*p* = 0.746), infection site (*p* = 0.990), and infected bacteria (*p* = 0.391) (*p* > 0.05). Compared with the no-recurrence group, education level (*p* = 0.010), smoking history (*p* = 0.044), hypoproteinemia (*p* = 0.024), open injury-related infection (*p* = 0.027), and combined flap surgery (*p* = 0.046) were significantly associated with recurrence (*p* < 0.05). These factors were further included in the binary logistic regression analysis. Results of the analysis showed that open injury-related infection (odds ratio [OR] = 35.698, 95% confidence interval [CI]: 5.997–212.495), *p* < 0.001) and combined flap surgery (OR = 41.408, 95% CI: 5.806–295.343, *p* < 0.001) were independent risk factors for the recurrence of osteomyelitis infection treated with antibiotic-loaded calcium sulfate combined with autologous iliac bone transplantation. Meanwhile, a high education level (OR = 0.009, 95% CI: 0.001–0.061, *p* < 0.001) was a protective factor for infection recurrence (Table 2).

**TABLE 1:** Single-factor analysis of infection recurrence.

Factor	Recurrence (n = 25)	No recurrence (n = 138)	*c* ^2^/t value	*p*-value
Age (years), mean ± SD	52.4 ± 15.7	50.7 ± 14.8	0.483	0.630
Sex, n (%)			<0.001	1.000^a^
Male	21 (84%)	115 (83.3%)		
Female	4 (16%)	23 (16.7%)		
Standard of culture, n (%)			9.293	0.010^a^
Primary school education and below	17 (68%)	49 (35.5%)		
Middle school	6 (24%)	61 (44.2%)		
University education and higher	2 (8%)	28 (20.3%)		
Smoking history, n (%)	15 (60%)	53 (38.4%)	4.059	0.044^b^
Complications, n (%)				
Diabetes	10 (40%)	39 (28.3%)	1.387	0.239^b^
Heart disease	3 (12%)	14 (10.1%)	<0.001	1.000^a^
Hypertension	5 (20%)	25 (18.1%)	<0.001	1.000^a^
Hypoproteinemia	13 (52%)	40 (29.0%)	5.109	0.024^b^
Previous surgery (≥2 times)	6 (24%)	26 (18.8%)	0.105	0.746^a^
Open injury-related infection, n (%)	19 (76%)	72 (52.2%)	4.872	0.027^b^
Infection sites, n (%)			0.885	0.990^a^
Tibia	13 (52%)	71 (51.4%)		
Femur	6 (24%)	33 (23.9%)		
Calcaneus	2 (8%)	13 (9.4%)		
Fibula	1 (4%)	6 (4.3%)		
Humerus	2 (8%)	8 (5.8%)		
Patella	1 (4%)	5 (3.6%)		
Ulna and radius	0	2 (1.4%)		
Bacterial species, n (%)			5.207	0.391^a^
*Staphylococcus aureus*	8 (32%)	74 (53.6%)		
Methicillin-resistant *Staphylococcus aureus*	4 (16%)	18 (13.0%)		
*Pseudomonas aeruginosa*	8 (32%)	26 (18.8%)		
Colibacillus	1 (4%)	7 (5.1%)		
*Staphylococcus epidermidis*	2 (8%)	9 (6.5%)		
No bacteria detected	2 (8%)	4 (2.9%)		
Combined flap surgery, n (%)	11 (44%)	34 (24.6%)	3.970	0.046^b^

^a^ Fishier exact test.

^b^ Chi-square test; SD: standard deviation.

**TABLE 2:** Multivariate analysis of infection recurrence.

Influencing factor	B	SE	Wald*c* ^2^	OR	95% CI	*p*-value
Standard of culture	−4.668	0.955	23.872	0.009	0.001–0.061	<0.001
Open injury-related infection	3.575	0.910	15.430	35.698	5.997–212.495	<0.001
Combined flap surgery	3.723	1.002	13.798	41.408	5.806–295.343	<0.001

OR: odds ratio, CI: confidence interval, SE, standard error.

## 4 Discussion

Antibiotic-loaded calcium sulfate combined with autologous iliac bone transplantation was used to treat localized osteomyelitis in one stage.

The current clinical treatment of osteomyelitis is primarily divided into staged treatment and one-stage treatment. The Masquelet technique serves as a representative method of staged treatment. Some researchers used the Masquelet technique to treat 424 patients with limb osteomyelitis, achieving a final infection control rate of 87.74% (372/424) (Wang et al., 2020). Compared with staged treatment, the time cycle of one-stage treatment offers a shorter time cycle, fewer number of operations, and lower infection control rates compared with its counterpart. Moreover, considering that osteomyelitis is mostly caused by severe trauma or fracture surgery, multiple operations in the early stage can significantly increase the patients’ economic burden. Conversely, one-stage treatment has a lower cost, alleviating the economic and psychological burdens faced by patients and their families (Ren et al., 2022). With advancements in surgical techniques and increasing patient demands, one-stage treatment of osteomyelitis is gaining popularity. In a previous study involving the primary treatment of 505 patients with chronic osteomyelitis, the use of muscle flaps, antibiotic-containing bioceramics or calcium sulfate, and bioactive glass for treatment yielded effective rates ranging from 87.5% to 94.2% (Pincher et al., 2019). Other studies used gentamicin-loaded calcium sulfate/nano-hydroxyapatite composites to treat osteomyelitis in one stage and achieved an infection control rate of 96% (96/100) (McNally et al., 2016). These findings suggest that the infection control rate of our one-stage treatment is comparable to, if not better than, than that of staged treatment.

In addition, the primary objective of osteomyelitis treatment is to control infection. A local drug delivery systems has been widely used in the control of infection in patients with osteomyelitis, as it can provide drug concentrations several times higher than the bacteriostatic concentration at the infection site. This approach reduces the systemic drug concentrations, thereby minimizing the occurrence of systemic adverse reactions (Zhang et al., 2010). Previously, the most commonly used local drug delivery system was antibiotic-loaded polymethyl methacrylate (PMMA), which can be combined with various antibiotics and released locally at the lesion site. However, it exhibits poor biodegradability, thus necessitating secondary surgery for removal after infection control. Although PMMA is suitable for the Masquelet technique of staged treatment, it is not applicable in one-stage treatment. Therefore, the antibiotic-loaded absorbable system with calcium sulfate as a typical example has emerged as the primary option in the first-stage treatment of osteomyelitis. This system has been used clinically to treat osteomyelitis and has achieved good results (Shi et al., 2022). Calcium sulfate is an excellent carrier of antibiotics. It can be combined with various antibiotics such as vancomycin, gentamicin, and tobramycin, and can completely release antibiotics after its degradation. Additionally, calcium sulfate has been reported to possess osteogenic properties, which can promote the endochondral ossification of the fracture end by stimulating growth factors such as vascular endothelial growth factor, transforming growth factor-1, and bone morphogenetic protein-2 (Ma et al., 2018). In addition, the implantation of calcium sulfate into the defect site not only prevents the inward growth of fibrous tissue, but also provides a bone-guided matrix for the growth of blood vessels and osteoblasts. As calcium sulfate gradually absorbs, new bone can replace the collagen scaffold, ultimately restoring the normal anatomical properties and structural characteristics (Kolk et al., 2012).

However, the problem of serous exudation during the process of calcium sulfate absorption cannot be ignored. Our previous study found that an increase in the amount of calcium sulfate implanted led to a higher incidence of serous exudation (Du et al., 2023). Jiang et al. used calcium sulfate alone to treat localized calcaneal osteomyelitis in a one-stage procedure. Although the infection eradication rate reached 85.3% (29/34), the nonserous exudation rate was as high as 32.35% (11/34) (Jiang et al., 2020). In addition, Rathbone CR et al. reported that high concentrations of local antibiotics can inhibit osteoblasts, which possibly contributes to nonunion after osteomyelitis treatment (Rathbone et al., 2011). In addition, when calcium sulfate was implanted at the bone defect site, although a new bone could gradually grow and replace the absorbed calcium sulfate, the degradation rate of calcium sulfate is much faster than the speed of new bone formation. The implantation of calcium sulfate alone or biological materials that did not match the speed of new bone formation could easily lead to nonunion of the defect (Peters et al., 2006). Some studies have used calcium sulfate hydroxyapatite composite materials to treat infectious bone defects, and the infection control rate reached 92.6%. However, the incidence of nonunion was approximately11% (Drampalos et al., 2020). Considering the possibility of complications such as serous exudation and nonunion caused by the simple implantation of calcium sulfate in the defect during the first-stage treatment, antibiotic-loaded calcium sulfate treatment combined with autogenous iliac bone transplantation was performed to utilize calcium sulfate solely as a carrier of sustained-release drugs. This approach reduced the total amount of implantation, thereby decreasing the incidence of complications. The incidence rates of aseptic serous exudation and nonunion in this study were 7.9% (13/163) and 0%, respectively, validating the effectiveness of this approach. Although the use of an autogenous iliac bone graft can reduce the incidence of these infection-related complications, this procedure can also cause complications in the bone removal area, including persistent chronic pain, hematoma formation, or anterolateral thigh hypoesthesia (Myeroff and Archdeacon, 2011). Although most of these complications typically resolve within a few months to half a year, it does not interfere with the final prognosis of patients. However, the discomfort experienced by patients during the recovery process warrants attention from clinical orthopedic surgeons (Kook et al., 2024). Therefore, during bone extraction, meticulous surgical technique is crucial to avoid organ damage; efforts should be made to preserve the iliac crest so that the pelvic belt is supported, thereby reducing the impact on the patient’s daily life (Huo et al., 2023).

In this study, antibiotic-loaded calcium sulfate combined with autologous iliac bone transplantation was used to treat limb-localized osteomyelitis in one stage. Compared with the traditional staged treatment, the treatment cycle was shortened, the number of operations was reduced, the treatment cost was reduced, and the infection control rate and fracture healing rate were not affected. Overall, this approach demonstrated favorable therapeutic efficacy. However, the associated risks cannot be ignored. The performance of autologous iliac bone transplantation in the first stage of treatment poses potential complications due to bone removal. If the infection recurs in the later stage, the remaining bone mass may be difficult to support the reconstruction of the bone defect, highlighting the importance of recognizing the risks of infection recurrence. Despite achieving positive outcomes in the treatment of localized osteomyelitis using this relatively novel method, the possibility of recurrence persists. Some scholars attribute infection recurrence to the type of bacteria and the site of infection, while others suggest a correlation with the number of operations and the method of fixation. However, no studies have specifically identified the risk factors of infection recurrence following this treatment for localized osteomyelitis. Our study addresses this gap by identifying the risk factors associated with infection recurrence and determining the patients suitable for one-stage treatment.

Risk factors for antibiotic-loaded calcium sulfate treatment combined with autogenous iliac bone transplantation as treatment for recurrent infection in patients with localized osteomyelitis.

The recurrence of osteomyelitis can be influenced by various factors, such as the cause of injury, the host’s immune status, type of bacteria, and condition of the skin and soft tissue. Some scholars have found that the risk factors for the recurrence of limb osteomyelitis infection treated with induced membrane technology include repeated surgery, post-traumatic osteomyelitis, and one-stage fixation (Wang et al., 2020). In addition, previous studies have shown that segmental bone defects, Gram-negative bacterial infections, and smoking are risk factors for infection recurrence in patients with osteomyelitis treated with antibiotic bone cement spacers (Wu et al., 2023). Different treatment methods have different risk factors for the recurrence of osteomyelitis infection. Our results suggest that combined flap surgery (OR = 41.408, 95% CI: 5.806–295.343, *p* < 0.001) and open injury-related infection (OR = 35.698, 95% CI: 5.997–212.495, *p* < 0.001) are independent risk factors for the recurrence of localized osteomyelitis infection treated with antibiotic-loaded calcium sulfate combined with autogenous iliac bone transplantation.

Previous studies have reported that good blood supply and skin and soft tissue coverage of the upper limbs are more successful than lower limb bone infections (Honda and McDonald, 2009). Wang et al. proposed that the high recurrence rate of tibial osteomyelitis could be attributed to poor blood supply and inadequate soft tissue coverage in the tibia (Wang et al., 2020). This aligns with the findings of our study. Our study found that patients undergoing combined flap surgery had a recurrence rate of 24.4% (11/45), which was higher than that of patients without skin and soft tissue involvement (11.9%, 14/118). Similarly, patients with open injury-related infections had a recurrence rate of 20.9% (19/91), which was higher than that of patients with closed injuries (8.3%, 6/72). This discrepancy can be attributed to several factors. First, early soft tissue coverage can improve the patient’s blood supply, provide nutritional support, promote local immune defense, and enhance antibiotic efficacy (McNally et al., 2017). On the contrary, patients who undergo combined flap surgery and develop open injury-related infections often present with varying degrees of skin and soft tissue damage, poor local blood supply, and incomplete pathogen eradication in the deep part of the lesion. After the host’s immunity decreases, the likelihood of infection recurrence significantly increases. Second, patients with open injury-related infections typically experience significant wound contamination and are prone to contracting multiple microbial infections (Tennent et al., 2018). In addition, patients with open injuries often endure extended hospital stays and are at higher risk of acquiring nosocomial infections caused by drug-resistant bacteria (Kay et al., 2014). These factors contribute to the difficulty of eliminating infections and heighten the likelihood of recurrence. Therefore, meticulous surgical planning, the active reconstruction of soft tissue defects, restoration of local blood supply, and focused attention on the treatment of open injuries are crucial strategies for controlling the recurrence of osteomyelitis. In addition, our regression model showed that a higher education level (OR = 0.009, 95% CI: 0.001–0.061, *p* < 0.001) was a protective factor against infection recurrence. This does not imply that individuals with higher education levels are immune to relapse. Individuals with higher education levels have a reduced likelihood of recurrence of infection compared with those with lower education levels. The author believes that individuals with low education levels often demonstrate lower compliance and may overlook or struggle to understand the postoperative precautions prescribed by doctors, thus affecting the prognosis of surgery. Therefore, for patients with lower education levels, clinicians should thoroughly explain all post-surgery instructions. Additionally, when patients are older adults or exhibit poor compliance, involving their spouses or children in the education process can help ensure comprehension and adherence to the recommended guidelines.

In addition, through a univariate analysis, a difference was found between smoking history (*p* = 0.044) and hypoproteinemia (*p* = 0.024). Binary logistic regression analysis showed that smoking history and hypoproteinemia were not independent risk factors for infection recurrence. However, smoking can affect the prognosis by impairing the immune status of patients and is an important risk factor for multiple bone infections (Lenguerrand et al., 2019). Hypoproteinemia reflects the nutritional status of patients. Malnourished patients often exhibit compromised immunity, delayed wound healing, and a high risk of infection recurrence. Another study highlighted hypoproteinemia as a risk factor for flap coverage necrosis (Keys et al., 2010). Therefore, more attention should be paid to patients with smoking history and hypoproteinemia. In summary, patients who have undergone combined flap surgery and those with open injury-related infections may not be suitable candidates for this surgical approach. On the contrary, patients with high education levels and strong compliance may benefit more from using this one-stage treatment.

This study has some limitations. First, this retrospective study was conducted in a single center and used a small sample size, potentially introducing bias into the data. Second, only one method was used to treat osteomyelitis, without providing a comparison to other techniques. Additionally, including all potential risk factors is challenging, and some important factors may be overlooked. Future studies should involve collaborating with medical centers in other regions to expand the sample size and include more risk factors for analysis.

## 5 Conclusion

Antibiotic-loaded calcium sulfate combined with autologous iliac bone transplantation is an effective method for the treatment of limb-localized osteomyelitis. Patients without previous combined flap surgery and non-open injury-related infections have a relatively low probability of recurrence of infection after treatment with this surgical method. Patients with smoking history and hypoproteinemia should pay attention to preventing the recurrence of infection after operation. Provided that implementation is feasible, offering more guidance tailored to patients with lower education levels and compliance could potentially yield positive outcomes in reducing the recurrence rate of infection.

## Data Availability

The original contributions presented in the study are included in the article/Supplementary material, further inquiries can be directed to the corresponding authors.
